# Comparison of the performance of the CareStart Malaria Pf/Pan Combo test and field microscopy in the diagnosis of *Plasmodium vivax* malaria in North Sumatera, Indonesia

**DOI:** 10.1186/s12936-022-04057-1

**Published:** 2022-01-29

**Authors:** Ayodhia Pitaloka Pasaribu, Irma Sari Nasution, Krisnarta Sembiring, Fahmi Fahmi, Syahril Pasaribu

**Affiliations:** 1 Department of Child Health, Medical Faculty, Universitas Sumatera Utara, Medan, 20155 Indonesia; 2 Medical Faculty, Universitas Sumatera Utara, Medan, 20155 Indonesia; 3 Department of Electrical Engineering, Universitas Sumatera Utara, Medan, 20155 Indonesia

**Keywords:** CareStart, Field microscopy, Malaria, Rapid diagnostic test, Indonesia

## Abstract

**Background:**

In areas where malaria is endemic and where trained microscopists are not available, rapid diagnostic tests (RDTs) are needed not only to allow prompt treatment without delay but also to prevent overdiagnosis and overtreatment based on clinical judgements that may lead to drug resistance. This study aimed to compare the performances of the CareStart Pf/Pan Combo test to field microscopy, which is considered to be the gold standard for malaria diagnosis.

**Methods:**

Any person with a fever or a history of fever within 48 h who came to the health centre was recruited for the study and tested both by the CareStart Pf/Pan test and by field microscopy. Sensitivity, specificity, positive predictive value, negative predictive value, and accuracy were analysed with both methods.

**Results:**

Two-hundred study participants were enrolled: 96 (48%) were found to be positive through microscopy, while 100 (50%) participants were found to be positive through RDT. The RDT produced four false-positive results. High sensitivity and specificity were observed for the CareStart Pf/Pan test (100 and 96.15%, respectively). The CareStart Pf/Pan test also showed excellent agreement with the field microscopy results.

**Conclusion:**

The Carestart Pf/Pan could be used as an alternative diagnostic test in malaria-endemic areas where facility for performing microscopy is not available.

## Background

Malaria remains a leading cause of morbidity and mortality worldwide, with an estimated 229 million new cases and 409,000 deaths annually [1]. As in other countries in Southeast Asia, Indonesia aims to achieve malaria elimination by 2030, and scaling up diagnostics and treatment interventions is currently essential [2]. In areas where malaria transmission has declined, accurate diagnosis is very important, not only for case management but also for disease surveillance [3, 4]. Clinical signs and symptoms of malaria are non-specific, which may result in overdiagnosis and inappropriate treatment using anti-malarial drugs that can lead to the selection of drug-resistant variants [4, 5]. The main strategy for malaria control of the World Health Organization (WHO) is to improve diagnosis by detecting parasites by either microscopy or by a rapid diagnostic test (RDT) [6]. Microscopy remains the gold standard for malaria diagnosis because it is inexpensive and can provide information on the specific parasite species and parasite density [7].

However, microscopy is time consuming and requires trained personnel, careful preparations and a constant supply of power for operating equipment, which is limited in most areas where malaria is endemic [8]. RDTs could be used as an alternative method for the diagnosis of malaria in areas lacking microscopes or trained technicians and allows for laypersons to conduct the test after limited training [9]. Over 60 different malaria RDTs have geographical areas [10, 11]. Factors such as ambient conditions, variability of the detected antigen and different parasite densities may influence the performance of the tests [10]. Several previous studies found that the CareStart Pf/Pan combo test has a higher sensitivity for *Plasmodium falciparum* than for *Plasmodium vivax* compared to microscopy [12, 13].

For *P. vivax*, pan-lactate dehydrogenase (pLDH) tests showed sensitivities ranging from 62.0 to 95.0% [12–14]. Polymerase chain reaction (PCR) allows low densities of malaria parasites to be detected in blood samples. It is important for epidemiological and clinical research especially for sub-microscopic recurrences, however, it should not be used in diagnosis and management as it is too sensitive [15].

This study was conducted to evaluate the performance of the CareStart RDT Pf/Pan combo test for the detection of *P. vivax* compared to field microscopy, which is the gold standard, in the Tanjung Leidong subdistrict, North Sumatra, Indonesia. This strategy is in line with the recent Malaria National Strategic Plan for 2020–2024 for Indonesia that recommends regular evaluations with RDTs within the country [16]. The hypothesis was that the CareStart Pf/Pan combo test has a comparable performance to field microscopy for the diagnosis of *P. vivax*.

## Methods

### Study area and design

**Fig. 1:**
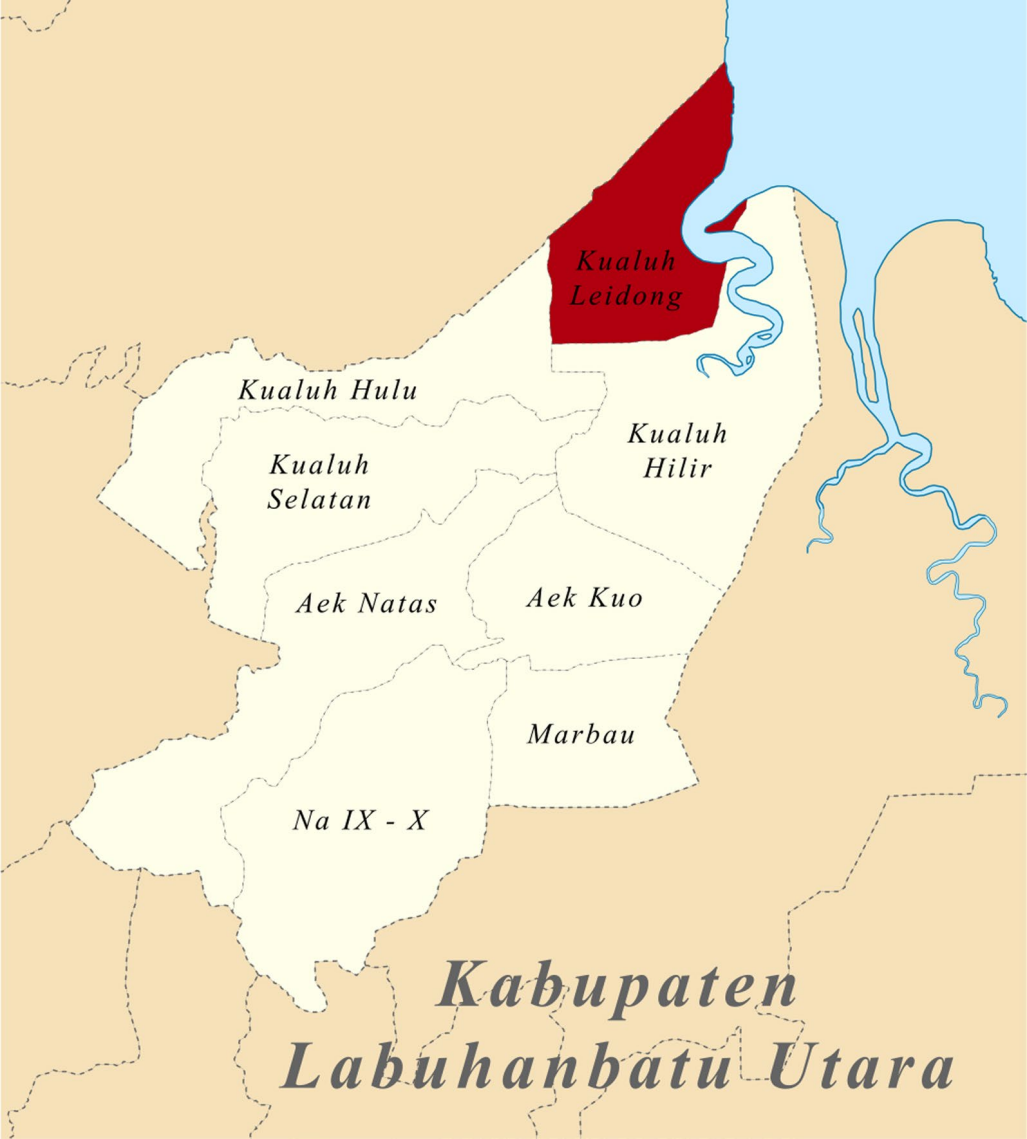
Map of the study site. https://commons.wikimedia.org/wiki/Category:Kualuh_Leidong,_Labuhanbatu_Utara

The cross-sectional study comparing RDT to field microscopy was conducted at Tanjung Leidong Health Centre in Kualuh Leidong District, North Labuhan Batu Regency, from July to December 2018, which was a peak transmission season for malaria in the area. The district covers an area of 394 sq km with a population of more than 29,552, the majority of which depends on fishing (Fig. 1) [17]. Tanjung Leidong Health Centre offers malaria diagnosis and treatment for free. *Plasmodium vivax* is the predominant vector species in this location.

### Sample size

To detect *P. vivax* with a sensitivity of 80% (alpha error 0.1, precision 5%), 83 blood smear-positive and 83 blood smear-negative samples were required. The detection of malaria-carrying species was limited to *P. vivax*.

### Study procedure

In inclusion criteria, any person with a fever or a history of fever within 48 h who visited the health centre was selected as a study participant. As for exclusion criteria, individuals who took anti-malarial drugs within the four weeks prior to the study or refused to participate were excluded. Both CareStart Pf/Pan combo tests and thick and thin blood smear microscopic examinations were simultaneously performed on samples obtained from each patient with acute febrile illness.

The diagnostic performance of the CareStart Pf/Pan combo test targeting the pLDH antigen was evaluated against the performance of thick blood-smear microscopy. Microscopy is still the gold standard for diagnosing malaria, thus under field condition it may miss some symptomatic cases, however it is still suitable for clinical diagnosis [15]. The health centre staff members were trained on selecting acute febrile cases and collecting samples for laboratory diagnosis. Explanations regarding the study were given to the participants before the samples were collected. Thick and thin blood smears were prepared on the same slide; the thin blood smears were fixed with methanol, and the thick smears were unfixed. Each slide was then stained with a 3% Giemsa solution for 45 min. All blood smears were examined microscopically under an oil immersion lens at 1000 ×  magnification. For positive smears, the numbers of parasites were counted against 200 white blood cells (WBCs) in thick smears or 500 WBCs for low-density infections. Parasite density was calculated assuming 8000 WBCs per µl (18) [18]. The thin smears for the positive samples were examined to confirm the species identification. All sample slides included in this study were examined by a single field microscopist who was blinded to the RDT results. All positive slides were sent to Eijkman Institute, Jakarta, for quality control. The RDT kit used was the CareStart Malaria HRP-2/pLDH (Pf/Pan) combo test (Access Bio, Belgium). This kit is a three-band RDT targeting HRP-2 and pan-pLDH.

The CareStart tests used in this study were donated by the Ministry of Health. Test kits were stored at room temperature and the expiration date was checked before use. The tests were performed according to the directions of the manufacturer. The CareStart tests were labelled with a patient identification number and date, and the results were recorded after 20 min. The presence of a unique pan-pLDH line is found when an individual is infected with one or more non-falciparum species. A test result without a control line was considered invalid, and the sample was retested. All the data were double-entered by study staff to maintain accuracy.

### Data analysis

Data were entered and analysed using STATA version 15.1 (Stata Corporation, College Station, TX, USA) after checking for completeness. Data were analysed for descriptive statistics, while results of both methods were compared using Cohen’s kappa co-efficient. The sensitivity, specificity, positive predictive value (PPV), and negative predictive value (NPV) were calculated. All statistical analyses were done at 95% level of significance.

## Results

During the study period, 207 patients with fever were screened and tested for malaria with the CareStart Pf/Pan test and field microscopy, among which 200 patients agreed to participate. The other seven patients refused to participate. More than one-half of the study participants were male (55.5%). By field microscopy, 96 (48%) study participants were found to be positive for *P. vivax* infection, while 100 (50%) tested positive with the CareStart Pf/Pan test. The only species detected in the positive blood smears and RDTs was *P. vivax*. The baseline characteristics are shown in Table 1.

**Table 1:** Baseline characteristics

Study participant characteristics (n = 200)	
Sex, n (%)	
Female	89 (44.5)
Male	111 (55.5)
Age classification, n (%)	
< 18 years	81 (40.5)
> 18 years	119 (59.5)
Mean hemoglobin level, g/dL (SD)	12.0 (1.83)
Median temperature, °C (range)	38.0 (36.4 – 40.2)
RDT result, n (%)	
Positive	100 (50.0)
Negative	100 (50.0)
Microscopic examination result, n (%)	
Positive	96 (48.0)
Negative	104 (52.0)
Parasitological characteristics (n = 96)	
Geometric mean asexual density parasite/µL (95% CI)	2717.47 (2143.39–3445.32)
Geometric mean gametocyte density/µL (SD)	2128 (1195.71)

Using microscopy as the gold standard test for detecting malaria by *P. vivax* infection, the sensitivity and specificity of the CareStart Pf/Pan combo test were 100% (95% CI 96.23–100) and 96.15% (95% CI 90.18–98.43), respectively. There were four (2%) discordant results obtained between field microscopy and the CareStart Pf/Pan test. All the discordant results were negative in the field microscopy test and positive in the CareStart Pf/Pan test. Unfortunately, the discordant samples were not tested to PCR as a confirmatory test that would help improve the reliability of the test.

The PPV and the NPV of the CareStart Pf/Pan test were determined to be 98% (95% CI 94.96–99.45) and 100%, respectively. The agreement of the results between the CareStart Pf/Pan tests and the field microscopy tests were excellent, with a kappa value of 0.96 (Table 2). The results of the quality control tests performed at the Eijkman Institute showed that 4 of the 96 field microscopy-positive samples were negative. The sensitivity of field microscopy compared to the Eijkman Institute readings was 97.87% (95% CI 92.52–99.74) and the PPV was 95.83% (95% CI 89.79–98.37). The readings at the Eijkman Institute differed from the field microscopy because of the level of expertise; the Eijkman Institute is a reference laboratory with expert readers, while in the field there was a basic reader.

**Table 2:** Performance of the CareStart Pf/Pan test compared to the gold standard, field microscopy

Field microscopy		CareStart Pf/Pan	
		Positive	Negative
	Positive	96	0
	Negative	4	100
	Sensitivity, % (95% CI)	100 (96.23–100)	
	Specificity, % (95% CI)	96.15 (90.44–98.99)	
	PPV, % (95% CI)	96 (90.18–98.40)	
	NPV, % (95% CI)	100 (N/A)	
	Accuracy, % (95% CI)	98 (94.96–99.45)	
	Cohen’s kappa (95% CI)	0.96 (0.92–0.99)	

**Fig. 2:**
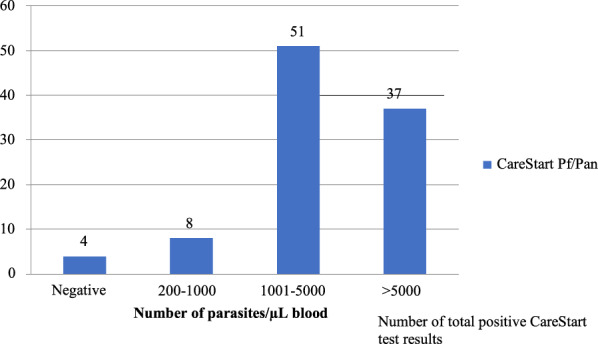
Number of positive CareStart Pf/Pan test results, categorized into different *P. vivax* densities

The CareStart Pf/Pan test revealed an increasing number of positive results with increasing levels of parasitaemia, with parasitaemia  > 1000/µl, accounting for 91.67% of all positive RDT results (Fig. 2).

## Discussion

RDTs for malaria diagnosis are vital to make prompt treatment decisions, especially at healthcare facilities that do not have trained microscopists [18, 19]. Even though a detailed assessment of the performance had been undertaken by WHO/Foundation for innovative diagnostic (FIND), it is important to evaluate the tests in different endemic area settings and as performed by health workers involved in the daily healthcare routine [20, 21]. In this study, the only malaria-causing parasite observed was *P. vivax.* This finding was similar to the study performed at the same site in 2013 [22] but differed from findings obtained in Nigeria, where *Plasmodium falciparum* was the only observed parasite [21]. The majority of the study participants in the present study were male and older than 18 years, which was similar to the cohort in previous studies conducted in Ethiopia and at the China-Myanmar border [23, 24].

A study performed in Korea found that one patient tested negative by RDT (SD BIOLINE), while the microscopy result was positive, which was a result similar to that of a study performed in Ethiopia with the CareStart combo test [25, 26]. These findings were different from those of the present study, where four study participants tested positive by the CareStart Pf/Pan test but negative by field microscopy. Several reasons for this result might be due to other infectious diseases or the presence of rheumatoid factors that were not assessed in this study [27]. Another reason could be attributable to pLDH production by plasmodial gametocytes, which may result in false-positive RDT results [28]. The overall diagnostic performance of the CareStart Pf/Pan test when compared to that of field microscopy was very good in the present study. The sensitivity and specificity of the CareStart Pf/Pan test in this study were 100 and 96.15%, respectively. These results were similar to several studies that were conducted in different areas [18, 19] but higher than reported studies conducted in Ethiopia [25].

Interestingly, the high sensitivity of the CareStart Pf/Pan test in this study was different to that in a study from Sumba, Indonesia, where the sensitivity of the test was only 13.6% [29]. The higher sensitivity might have been the result of the high number of parasites found in the current study’s samples (> 200 parasites/µL). The high positive and negative predictive values in this study were similar to the findings of Feleke and Moges obtained in northwestern Ethiopia [25, 30]. The sensitivity could not be categorized based on different parasite densities because the overall sensitivity was 100%; however, an increasing number of positive RDT results was found with increasing parasitaemia. Several studies using the CareStart RDT reported higher sensitivity with higher parasitaemia [23, 24] but another study performed in South Korea presented different results [31]. This study also observed excellent agreement between the CareStart Pf/Pan test results and the field microscopy results, which was similar to other studies [24].

Limitations of this study included the cross-sectional design that limited the detection of sub-microscopic parasites that may have been found in a follow-up test. In the present study, only the CareStart Pf/Pan test results were compared with field microscopy results; however, the true burden of sub-microscopic infection would have been better determined by polymerase chain reaction (PCR) and new diagnostic tests should have the ability to detect sub-microscopic infections to achieve malaria elimination [32].

Nevertheless, the comparison of RDT and microscopy results for malaria diagnosis is seldom conducted in Indonesia, and the study results are important because they are in line with the current Malaria National Strategic Plan for 2020–2024.

## Conclusion

The CareStart Pf/Pan test showed very good sensitivity and specificity with excellent agreement with the results obtained with use of the gold standard, field microscopy. This result provides important information to the Ministry of Health.

## Data Availability

The data used to support the findings of this study are available from the corresponding author upon request.

## Abbreviations

HRP: Histidine rich protein
pLDH: Pan-lactate dehydrogenase
PCR: Polymerase chain reaction
RDT: Rapid diagnostic test
WBC: White blood cell
WHO: World Health Organization
